# Variability in human plasma volume responses during high‐altitude sojourn

**DOI:** 10.14814/phy2.14051

**Published:** 2019-03-28

**Authors:** Andrew J. Young, James P. Karl, Claire E. Berryman, Scott J. Montain, Beth A. Beidleman, Stefan M. Pasiakos

**Affiliations:** ^1^ U.S. Army Research Institute of Environmental Medicine Natick Massachusetts; ^2^ Oak Ridge Institute for Science and Education Belcamp Maryland

**Keywords:** Acclimatization, hemoconcentration, hypoxia, intravascular volume

## Abstract

When sea‐level (SL) residents rapidly ascend to high altitude (HA), plasma volume (PV) decreases. A quantitative model for predicting individual %∆PV over the first 7 days at HA has recently been developed from the measurements of %∆PV in 393 HA sojourners. We compared the measured %∆PV with the %∆PV predicted by the model in 17 SL natives living 21 days at HA (4300 m). Fasting hematocrit (Hct), hemoglobin (Hb) and total circulating protein (TCP) concentrations at SL and on days 2, 7, 13, and 19 at HA were used to calculate %∆TCP and %∆PV. Mean [95%CI] measured %∆PV on HA2, 7, 13 and 19 was −2.5 [−8.2, 3.1], −11.0 [−16.6, −5.5], −11.7 [−15.9, −7.4], and −16.8 [−22.2, −11.3], respectively. %∆PV and %∆TCP were positively correlated (*P* < 0.001) at HA2, 7, 13, and 19 (*r* ^2^ = 0.77, 0.88, 0.78, 0.89, respectively). The model overpredicted mean [95% CI] decrease in %∆PV on HA2 (−12.5 [−13.9, −11.1]) and HA7 (−21.5 [−23.9, −19.1]), accurately predicted the mean decrease on HA13 (−14.3, [−20.0, −8.7]), and predicted a mean increase in %∆PV on HA19 (12.4 [−5.0, 29.8]). On HA2, 7, 13, and 19 only 2, 2, 6, and 1, respectively, of 17 individual measures of %∆PV were within 95% CI for predicted %∆PV. These observations indicate that PV responses to HA are largely oncotically mediated, vary considerably among individuals, and available quantitative models require refinement to predict %∆PV exhibited by individual sojourners.

## Introduction

Soon after people who live at sea level (SL) ascend to high altitude (HA), their plasma volume (PV) begins to decrease, causing hematocrit (Hct) and hemoglobin concentration (Hb) to increase. This hemoconcentration increases arterial oxygen carrying capacity (Sawka et al. 2000) thereby satisfying muscle oxygen requirements during submaximal aerobic exercise with a lower cardiac output (and less cardiovascular strain) that would be possible without this physiological adjustment. Collectively, these physiological adjustments developing early during altitude acclimatization contribute to the improvement in aerobic exercise endurance observed during the 1st or 2nd week of sojourns at HA (Maher et al. 1974; Horstman et al. 1980; Pandolf et al. 1998). However, these hematological adjustments also have the potential to influence the parameters used to generate the Athlete Biological Passport (Sottas et al. 2011) for athletes incorporating HA training in their conditioning program, such that a positive indication for blood doping might be calculated. Therefore, there is a need to reliably predict what an individual's “normal” PV response would be during sojourn at HA. Further, since PV changes and other fluid shifts between body compartments may influence performance (Robertson et al. 1982) at HA and/or susceptibility to HA illnesses (Gatterer et al. 2013), this prediction capability may be applicable and has utility for military personnel and others sojourning at altitude.

A quantitative model has recently been described (Beidleman et al. 2017) for predicting the magnitude of PV changes (%∆PV) that a HA sojourner would experience. The model was developed by regressing parameters describing the altitude ascended, duration of altitude exposure, age and sex of the altitude sojourner, and the interactions among those parameters on the %∆PV observed in 393 unacclimatized, well‐nourished men and women who had rapidly ascended to altitudes ranging from 2500 to 4500 m and remained for periods of 2 h to 7 days (most for 2–36 h) (Beidleman et al. 2017). The developers intended that the model would be used to describe the “normal” PV responses exhibited by SL athletes while training at HA, thus allowing identification of abnormal or unexpected responses that might have implications for performance or health during HA sojourns, and/or possibly indicating the use of banned blood doping practices (Beidleman et al. 2017). However, while that model was internally cross‐validated using a boot‐strapping resampling process, it has not been externally validated against %∆PV measured in an independent sample of HA sojourners. Further, real‐world sojourns at HA often last longer than 7 days, and sojourners often experience energy deficit and body mass losses (Rose et al. 1988; Butterfield 1996; Pasiakos et al. 2017). If the model could be shown reliable for predicting responses to altitude under those conditions, its utility would be extended.

The model for predicting %∆PV during HA sojourns (Beidleman et al. 2017) does not include a parameter accounting for changes in total circulating protein (∆TCP), but considerable evidence indicates that the PV reduction during HA sojourns is largely oncotically mediated. In SL residents who ascend to HA, total circulating protein (TCP) decreases, and the magnitude of the ∆TCP strongly correlates with the magnitude of the concomitant ∆PV (Sawka et al. 1996). The decrease in TCP results from protein translocation from intravascular to extravascular space (Surks 1966; Westergaard et al. 1970). Changes in the rate of breakdown of some plasma protein have also been suggested to contribute to the decrease in TCP (Westergaard et al. 1970).

Recently, we conducted a study investigating the effects of varied levels of dietary protein intake on body composition changes experienced by 17 healthy SL residents who ascended to 4300 m and remained living for 21 consecutive days at that altitude in negative energy balance (Berryman et al. 2018). Our study participants (Berryman et al. 2018) had similar characteristics to the population used to develop the recently published quantitative model (Beidleman et al. 2017), providing an opportunity to compare the %∆PV between SL and HA measured in an independent population of participants to the %∆PV predicted for these same participants using the model (Beidleman et al. 2017). Thus, the aim of this study was to determine how accurately the recently published quantitative model (Beidleman et al. 2017) for predicting changes in PV of well‐nourished SL residents during the first 7 days after ascent to HA would predict changes in PV exhibited by SL residents during a 21‐days high‐altitude sojourn, under real‐world conditions of energy deficit and weight loss. Since our participants were all men and they resided at a single altitude, effects of variations of those parameters on model predictions were avoided. We hypothesized that %∆PV measured in our participants during the first 7 days at HA would be accurately predicted by the model, but changes measured after 7 days at altitude would not be as well predicted, as the effects of altitude acclimatization and negative energy balance on factors modulating Hb, Hct, and PV at altitude accumulated in our participants.

## Methods

### Participants and study design

The analyses reported herein were included as secondary objectives in a randomized, controlled feeding study that examined the effects of manipulating dietary protein on fat‐free mass during HA sojourn (Berryman et al. 2018). Participants were 17 young, healthy, physically active male SL natives (Table 1). The study was approved by the Institutional Review Board at the U.S. Army Research Institute of Environmental Medicine (Natick, MA), conducted May–Aug 2016, and registered on www.clinicaltrials.gov as NCT02731066. Investigators adhered to the policies for the protection of human participants as prescribed by Army Regulation 70‐25, and the research was conducted in adherence with the provisions of 32 CFR Part 219.

**Table 1:** Prestudy characteristics of participants

Characteristic	Mean ± SD
Age, y	23.4 ± 5.6
Height, cm	177 ± 7
Weight, kg	81.9 ± 13.9
Body mass index, kg/m^2^	26.2 ± 3.6
Body fat, %	22.7 ± 6.1
V̇O_2_peak, L/min	4.17 ± 0.65

The study design has been reported in detail, previously (Berryman et al. 2018; Young et al. 2018). Briefly, the study consisted of two phases conducted over 43 consecutive days. Body mass was measured in undergarments, upon waking (overnight fast, ≥8 h), and after voiding, using a calibrated digital scale (Befour model PS6600; Befour Inc., Saukville, WI) to the nearest 0.1 kg daily, throughout the study. During the first 21 days (phase 1, SL), participants resided at SL, consumed a self‐selected, weight‐maintaining diet, maintained habitual exercise routines, and were free living but visited the laboratory daily. Body composition was determined at SL (day 0) using dual energy X‐ray absorptiometry (DEXA, DPX‐IQ, GE Lunar Corporation, Madison, WI). On SL day 21, participants were flown from Boston, MA to Denver, CO where they were placed on supplemental oxygen until being driven to the summit of Pikes Peak, CO (4300 m) the following morning (HA day 0) where they resided for the next 22 days at the U.S. Army Research Institute of Environmental Medicine Maher Memorial Laboratory (phase 2; HA). During HA, participants were under constant supervision, consumed a controlled and measured diet, and engaged in prescribed physical activity.

### Study diets

The diets have been described in detail, previously (Berryman et al. 2018). Briefly, beginning on HA1, the first full day of residence at 4300 m, and continuing until they completed the HA phase of the study, participants consumed a controlled diet designed to induce weight loss which is common during HA sojourn (Pasiakos et al. 2017). Computer‐generated randomization was used to assign participants to consume a diet containing either a standard‐protein (SP; 1.1 ± 0.2 g/kg/d) or higher‐protein (HP; 2.1 ± 0.2 g/kg/d), carbohydrate‐matched diet during HA. Water and noncaloric, noncaffeinated sodas were allowed ad libitum.

### Blood biochemistries

Blood samples were collected at SL (SL day 6), and on the 2nd, 7th, 13th and 19th full day of residence at 4300 m (HA2, HA7, HA13, and HA19, respectively). All blood samples were obtained by venipuncture after ≥ 20 min of seated rest, and following a >10 h fast. Hct and Hb concentrations were determined using an automated analyzer (i‐STAT™, Abbott Point of Care Inc., Princeton, NJ). Serum protein and osmolality were determined using an automated analyzer (Beckman Coulter DXC 600 Pro, Brea, CA), with osmolality calculated from sodium (Na), glucose (glu) and blood urea nitrogen (BUN) concentrations [Osm = (1.86*Na) + (Glu/18) + (BUN/2.8) + 9] (Dorwart and Chalmers 1975).

### Calculation of vascular volume changes

Each participant's lean body mass measured on SL day 0 was used to estimate their total blood volume at SL using the equation developed by Sawka et al. (1992). The Hct measured on SL day 6 was used to calculate the SL erythrocyte volume, and the corresponding PV was calculated as the difference between total blood and erythrocyte volumes. The % ∆PV on HA2, HA7, HA13, and HA19 relative to PV at SL was estimated from the differences in Hct and Hb concentrations using the Dill and Costill equation (Dill and Costill 1974). The absolute PV (and the absolute ∆PV) at each of the test days at HA was then calculated using the calculated absolute PV at SL, adjusted for %∆PV on that test day. TCP was calculated as the product of serum protein concentration and absolute PV for each test day, and %∆TCP was calculated as the difference between TCP at SL and TCP at each test day at HA, expressed at a % of the SL TCP.

### Data analyses and statistical tests

Statistical analyses were performed using SPSS v.24. All data were checked for adherence to model assumptions and for outliers. All tests were two‐sided and statistical significance was set at *P* ≤ 0.05. A general linear model (i.e., repeated measures ANOVA) without covariates was used to examine main effects of time, diet, and their interactions on Hct, Hb, TCP, osmolality, and the vascular volumes. No main or interaction effects of dietary protein level were found on any parameter, so diet was removed as a factor and the analyses rerun. When significant main effects of time were observed, post hoc comparisons were conducted and *P*‐values were adjusted using the Bonferoni correction.

The model developed by Beidleman et al. (2017) predicts %∆PV as follows:

$$\%\Delta \text{PV}=0.05-1.93\left(T\right)+0.27{\left(T\right)}^{2}-2.82\left(A\right)-0.58\left(A\right)\left(T\right)-0.82\left(S\right)+0.73\left(S\right)\left(T\right)+0.064\left(A\right)\left(Y\right),$$

where *T* is time at altitude (days), *A* is altitude (km), *S* is sex (male = 0, female = 1), and *Y* is age (years). The predicted % ∆PV was calculated for each of our participants using the same value for altitude, 4.04 km [that is the “physiological” altitude at the Pikes Peak Laboratory based on usual barometric pressures recorded, and this was the altitude used by the model developers (Beidleman et al. 2017) for data collected at the Pikes Peak laboratory], and each individual participant's age and sex. We ran the prediction four times, varying the time at altitude value as appropriate to predict % ∆PV on HA2 (*T* = 2), HA7 (*T* = 7), HA13 (*T* = 13), and HA19 (*T* = 19). The upper and lower limits of the 95% confidence interval were also calculated for the model predictions at each time point, over the age range that encompassed our participants. Finally, the model was used to predict % ∆PV at each of those times for the participant group as a whole, using mean age of the participant group as the age value. The individual and group mean values of the measured % ∆PV were then compared to the upper and lower limits of the 95% confidence interval for the predicted % ∆PV, to determine whether they fell within those limits.

## Results

**Figure 1:**
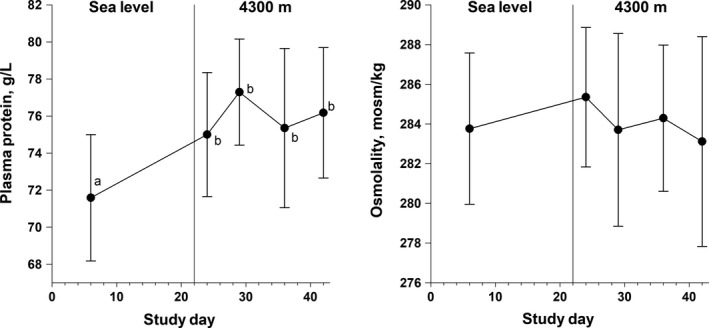
Mean ± SD of plasma protein concentration (Panel A) and osmolality (Panel B) measured in 17 male sea‐level residents at sea level and after 2, 7, 13, and 19 days of continuous residence at high altitude (4300 m). Means not sharing the same superscript are significantly different (main effect of day, *P* ≤ 0.001 and pairwise comparisons, all *P* ≤ 0.008).

**Figure 2:**
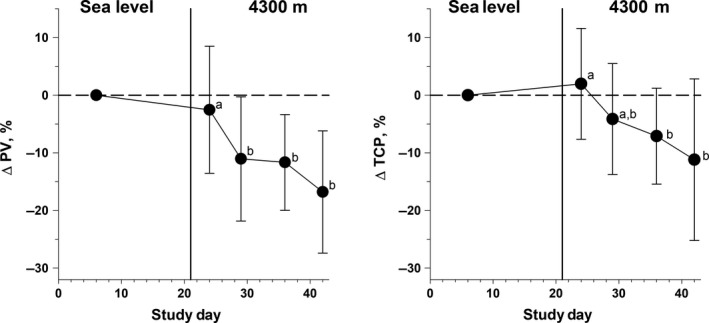
Mean ± SD of change in plasma volume (%∆PV) and total circulating protein (%∆TCP) measured in 17 male sea‐level residents after 2, 7, 13, and 19 days of continuous residence at high altitude (4300 m). Means not sharing the same superscript are significantly different (main effect of day, *P* ≤ main effect of day 0.001 and pairwise comparisons all *P* ≤ 0.045).

Both Hct and Hb increased (main effect of time, *P* ≤ 0.001) during the altitude sojourn (Table 2). The pairwise differences between SL and HA2 were not significant, but values at HA7, HA13, and HA19 were all greater (*P* < 0.05) than at SL. Plasma protein concentration (Fig. 1A) increased (main effect of time, *P* ≤ 0.001) with ascent from SL to HA, with the difference significant at HA2 and thereafter, but protein concentrations did not differ among HA2, HA7, HA13, and HA19. Plasma osmolality (Fig. 1B) did not differ between trials (main effect of time, *P* = 0.51). Blood, erythrocyte, and PV at SL were 4.97 ± 0.65 L, 2.20 ± 0.32 L, and 2.78 ± 0.39 L, respectively (Table 3). PV (Fig. 2A) and TCP (Fig. 2B) both decreased after ascent from SL to HA (Fig. 2, main effect, *P* ≤ 0.01) with the difference becoming larger as the sojourn continued. As indicated by the large standard deviations, there was considerable variability in these responses among participants.

**Table 2:** Hematocrit (Hct) and hemoglobin (Hb) concentrations at sea level (SL) and high altitude (HA)

	Day	Mean	95% Confidence interval for mean		Standard deviation
			Lower bound	Upper bound	
Hct, %	SL6	44.12^a^	42.74	45.50	2.69
	HA2	44.88^a^	43.46	46.30	2.76
	HA7	47.18^b^	45.75	48.60	2.77
	HA13	47.29^b^	46.12	48.47	2.28
	HA19	48.88^b^	47.48	50.29	2.74
Hb, g/100 mL	SL	15.00^a^	14.52	15.48	0.93
	HA2	15.26^a^	14.78	15.75	0.90
	HA7	16.05^b^	15.56	16.53	0.90
	HA13	16.08^b^	15.68	16.48	0.61
	HA19	16.61^b^	16.13	17.08	0.87

Hematocrit (Hct) and hemoglobin (Hb) at SL and HA (4300 m) over a 19 day sojourn (main effect of day, *P* ≤ 0.01). Means not sharing the same superscript are significantly different (pairwise comparisons, *P* ≤ 0.04).

**Table 3:** Vascular volumes at sea level

Participant	Lean mass^1^, kg	Blood^2^, L	Hematocrit^3^, %	Erythrocyte^4^, L	Plasma^5^, L
1	51.4	4.28	41.00	1.76	2.53
2	70.9	5.69	42.00	2.39	3.30
3	78.5	6.24	45.00	2.81	3.43
4	60.1	4.91	44.00	2.16	2.75
5	62.8	5.11	45.00	2.30	2.81
6	59.2	4.85	45.00	2.18	2.67
7	57.8	4.75	47.00	2.23	2.52
8	52.0	4.33	43.00	1.86	2.47
9	51.2	4.27	43.00	1.84	2.44
10	65.8	5.32	40.00	2.13	3.19
11	48.2	4.06	47.00	1.91	2.15
12	56.9	4.68	49.00	2.29	2.39
13	70.4	5.65	46.00	2.60	3.05
14	50.6	4.22	41.00	1.73	2.49
15	59.3	4.85	45.00	2.18	2.67
16	72.8	5.83	47.00	2.74	3.09
17	68.3	5.50	40.00	2.20	3.30

^1^Lean mass determined by DEXA on sea level day 0; ^2^total blood volume = 0.072 (lean mass) + 0.584 (Sawka et al. 1992); ^3^hematocrit measured on sea level day 6; ^4^erythrocyte volume = blood volume (hematocrit/100); ^5^plasma volume = blood volume–erythrocyte volume.

**Figure 3:**
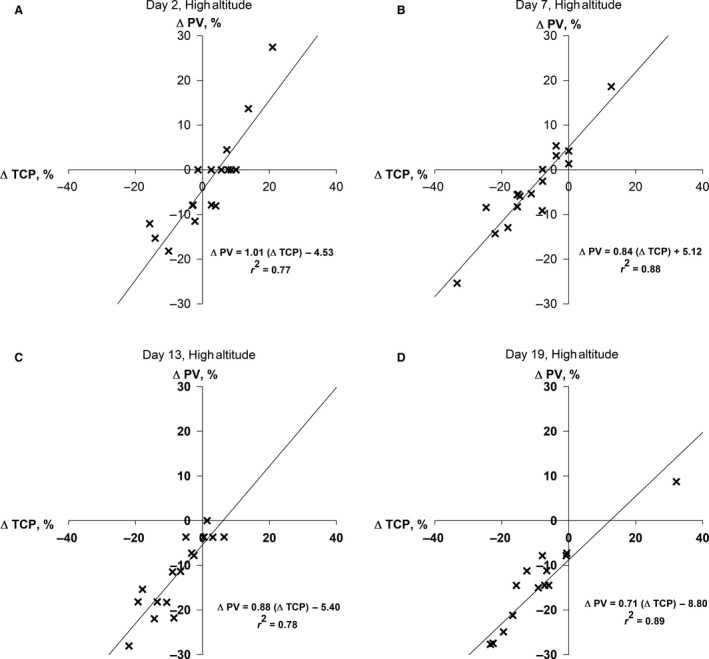
Individual changes in plasma volume (%∆PV) plotted as a function of the concomitant changes in total circulating protein (%∆TCP) measured in 17 male sea‐level residents after 2, 7, 13, and 19 days (Panels A, B, C and D, respectively) of continuous residence at high altitude (4300 m).

The variability in the %∆PV among the participants was not significantly correlated with absolute plasma volume at sea level (data not shown), but was strongly correlated with the variability in the %∆TCP (Fig. 3) at HA2, 7, 13 & 19 (*r* ^2^ = 0.77, 0.88, 0.78, 0.89, respectively; *P* ≤ 0.01). As reported in detail elsewhere (Berryman et al. 2018), participants total body mass loss was 7.9 ± 1.9 kg during the 21‐day residence at HA, but the absolute changes in body mass (relative to SL) on HA2, HA7, HA13, and HA19 were not significantly correlated with the corresponding %∆PV (*P* ≥ 0.13) or %∆TCP (*P* ≥ 0.21) on those days.

**Figure 4:**
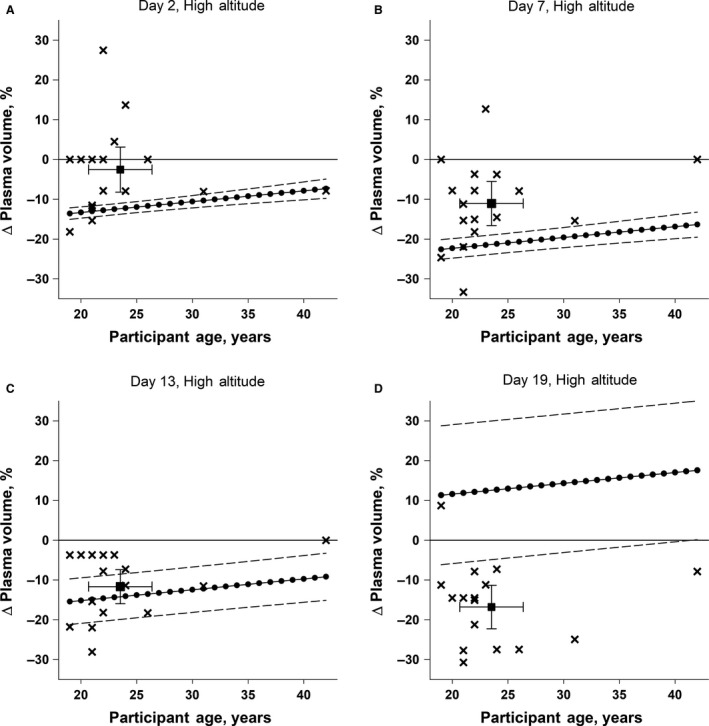
Individual (X) and mean ± 95% CI (filled square) of the % changes in plasma volume measured in 17 male sea‐level residents after 2, 7, 13, and 19 days (Panels A, B, C, and D, respectively) of continuous residence at high altitude (4300 m) plotted as a function of the participants’ ages. For each year of age across the age range of the participants, the model‐predicted (Beidleman et al. 2017) change in plasma volume is also plotted (filled circles), with upper and lower bounds of 95% CI of the model predictions indicated (dashed lines).

The model overpredicted mean [95% CI] decrease in %∆PV on HA2 (−12.5 [−13.9, −11.1]) and HA7 (−21.5 [−23.9, −19.1]), accurately predicted the mean decrease on HA13 (−14.3, [−20.0, −8.7]), and predicted an increase % ∆PV on HA19 (12.4 [−5.0, 29.8]), when mean measured % ∆PV still indicated a decrease in PV relative to SL (Fig. 4). Further, only 2, 2, 6, and 1 of 17 individual measures of % ∆PV on HA2, 7, 13 and 19, respectively, were within 95% CI for predicted %∆PV.

## Discussion

The primary aim of this study was to determine the extent to which a recently published quantitative model (Beidleman et al. 2017) for predicting changes in PV of well‐nourished SL residents during the first 7 days following ascent to HA accurately predicted the changes in PV exhibited by SL residents on the 2nd, 7th, 15th and 19th days of a high‐altitude sojourn, under real‐world conditions of progressively accumulating energy deficit and weight loss (Butterfield 1996). Our primary findings indicate that the PV changes experienced by our participants exhibited much greater inter‐individual variability than was predicted by the model, and the variability in the PV responses at HA appears to be mediated by changes in oncotic pressure associated with changes in TCP. These findings indicate that the model does not reliably predict the PV responses of individual HA sojourners, either during the first 7 days at altitude, or for longer sojourns, suggesting that the model does not account for all factors modulating ∆PV in response to hypoxia.

The model (Beidleman et al. 2017) did not reliably predict PV responses of individual sojourners who had been at HA longer than 7 days, the maximum sojourn duration for subjects whose data were used to develop the model. The individual %∆PV values of 11 of 17 and 16 of 17 participants in our study fell outside the 95% confidence interval for the predicted values on HA13 and HA19, respectively. This was not too surprising given that the model's developers had acknowledged that the quadratic equation used in their model was applicable to only the first 7 days at HA (Beidleman et al. 2017). However contrary to our hypothesis, the model (Beidleman et al. 2017) also did not reliably predict PV changes exhibited by our participants after 2 and 7 days of HA sojourn either, durations that are within the ranges of exposure durations for the participants whose data were used to develop the model. On both HA2 and HA7, individual %∆PV values of 15 of 17 participants in our study fell outside the 95% confidence interval for the predicted values. The lack of agreement between observed and predicted changes in PV at these shorter exposure durations may reflect the same limitation as the discrepancy observed and predicted changes at the longer exposure durations. Specifically, hypoxic exposure durations were only 2–36 h for well over half of the subjects whose data were used to develop the prediction model (Beidleman et al. 2017). Therefore, the parameter estimates developed for the model equation (Beidleman et al. 2017) may be more influenced by responses to relatively short hypoxic exposures (i.e., ≤36 h), making the model inherently less reliable for exposures ≥36 h). These findings indicate that the model needs to be refined by incorporating more data from longer altitude sojourns.

In agreement with previously reported findings (Sawka et al. 1996), we observed that at in all four trials during the 19‐day sojourn at HA, the change in PV (relative to SL) in our participants was highly correlated with the concomitant change in TCP. The *r* ^2^ values for the relationship indicated that 80–90% of the variation in the change in PV between SL and HA were accounted for by variation in the TCP content. The exact mechanism by which hypoxic exposure mediates changes in TCP is not entirely clear. However, pulmonary vasculature is known to become more permeable to proteins at HA (Bartsch et al. 2005), so probably other vascular regions do as well. Further, Westergaard et al. (1970) observed that plasma albumin synthesis and breakdown rates remained unchanged in SL residents living at 3450 m for 8 days, but albumin content shifted from intravascular to extravascular space, while at the same time the rate of IgG breakdown was increased at HA. More recently, we have observed that whole body protein synthesis is decreased during chronic HA exposure (Berryman et al. 2018). Thus, both decreased synthesis of plasma protein and leakage of plasma protein into the extravascular space could be contributing to a decrease in TCP during hypoxic exposure. If these mechanisms could be better characterized and quantified, perhaps a parameter describing that aspect of the PV response to altitude exposure could be devised and added to model to improve its reliability for predicting individual PV changes during altitude sojourns.

The possibility that certain elements of our research approach influenced our findings needs to be considered. Our participants were part of a larger study evaluating the effects of high versus moderate levels of dietary protein content during energy deficit while sojourning at HA. Therefore, the possibility that the energy deficit or variation in dietary protein intake may have influenced the regulation of PV during the altitude sojourn cannot be entirely ruled out. However, such an effect seems unlikely since, as described earlier, there were no effects of dietary protein on any measured or derived parameter, and no correlations were observed between changes in body mass (compared to SL) at any of the four altitude trials and the changes in PV and TCP. Further, the dietary manipulations began on HA1, so any undetected effects of diet on our results would likely have been minimal on HA2. Also, while we encouraged participants to drink frequently and provided easy access to ample amounts of flavored beverages and water, we did not measure changes in total body water during the altitude sojourn. Nevertheless, plasma osmolality remained unchanged from SL values throughout the altitude sojourn, suggesting that the participants were not dehydrated. Finally, the changes in PV, from which the changes in TCP were derived, were calculated using the Dill‐Costill equation (Dill and Costill 1974). This approach, which is similar to the approach used to assess plasma volume changes in the altitude sojourners studied by the developers of the model we were evaluating, assumes that total erythrocyte volume remains constant throughout the study. We have previously demonstrated that assumption is valid for at least the first 13 days of altitude acclimatization at 4300 m (Sawka et al. 1996). Moreover, since the maximal rate of erythrocyte volume expansion is 50 mL/week (Berglund and Ekblom 1991), erythrocyte volume in our participants (Table 3) could have increased no more than about 2% per week in our participants. Expansion of red cell mass of that magnitude would result in even smaller decreases in plasma volume on HA7, HA13, and HA19 than indicated by use of Dill‐Costill equation, and the agreement between observed and model‐‐predicted plasma volume changes would not be improved. Therefore, we believe use of the Dill‐Costill equation (Dill and Costill 1974) does provide valid estimates of PV changes during our study.

The model that we investigated was developed to predict the “normal” PV response to HA (Beidleman et al. 2017). Therefore, it is appropriate to consider to what degree the PV responses that we observed reflect “normal” HA responses. Recently, Siebenmann et al. (2017) reviewed the reduction in PV observed at HA in 21 studies. The mean PV reductions exhibited by our group of participants on HA7, HA13, and HA19 (11–17%) appear similar in magnitude to studies of similar exposure durations at altitudes >4000 m [Fig. 1A in Siebenmann et al. (2017)]. On the other hand, the mean reduction in PV exhibited by our participants on HA2 (2.5%) is somewhat less than observed in some, but not all of the studies reviewed by Siebenmann et al. (2017) that reported changes during the first 1–2 days at altitudes >4000 m. This might raise the question of whether our participant pool might have included a high prevalence of abnormal responders. However, we do not believe this is likely, since the mean PV changes (~8%) observed on the 1st day at Pikes Peak in control participants in a previous study conducted by Sawka et al. (1996) were within the 95% confidence interval of the mean PV reduction observed in the current study. Further, Sawka et al. (1996) observed that five of 16 participants exhibited no change in PV on the 1st day after arriving at Pikes Peak, whereas in our study six of 17 participants exhibited no change on the 2nd day at Pikes Peak. One factor that might account for the somewhat smaller PV reductions observed by Sawka et al. (1996) and in the current study is the physical activity of the subjects. Physical activity levels of participants in both the study by Sawka et al. (1996) and our current study (Berryman et al. 2018) were higher at HA than their habitual physical activity levels at SL. As discussed by Siebenmann et al. (2017), two factors appear to modulate the reduction in plasma volume at high altitude: (1) changes in oncotic pressure (as a result of changes in TCP) and (2) a hormonally mediated diuresis that increases loss of body water. Siebenmann et al. (2017) suggest that in HA sojourners who maintain high levels of physical activity, exercise may activate the renin‐angiotensin‐aldosterone and ADH systems such that diuresis is prevented or at least limited. In that case, only changes in oncotic pressure would be acting to reduce plasma volume, and the magnitude of the reduction might not be as pronounced as when hormonally mediated diuresis is contributing. Therefore, the variation in PV responses exhibited by our physically active participants at HA is likely to represent normal variation.

In conclusion, PV changes exhibited by sea‐level residents during sojourn at HA vary considerably between individuals. The changes in PV experienced by physically active people during high‐altitude sojourns are likely to be mediated primarily by changes in oncotic pressure, since the majority of the variability in the PV response to HA was accounted for by variability in the concomitant changes in TCP. Quantitative models currently available for predicting individual PV changes at HA (Beidleman et al. 2017) require further refinement to improve capability for identifying whether an individual's response is “normal” or indicative of the use of banned blood doping practices.

## Conflict of Interest

The investigators adhered to the policies for protection of human subjects as prescribed in Army Regulation 70‐25, and the research was conducted in adherence with the provisions of 32 CFR part 219. The opinions or assertions contained herein are the private views of the authors and are not to be construed as official or as reflecting the views of the Army or the Department of Defense. Any citations of commercial organizations and trade names in this report do not constitute an official Department of the Army endorsement of approval of the products or services of these organizations. The authors declare that they have no conflicts of interest.
